# Assessment of Flow through Microchannels for Inertia-Based Sorting: Steps toward Microfluidic Medical Devices

**DOI:** 10.3390/mi11100886

**Published:** 2020-09-24

**Authors:** Rucha Natu, Suvajyoti Guha, Seyed Ahmad Reza Dibaji, Luke Herbertson

**Affiliations:** Division of Applied Mechanics, Office of Science and Engineering Laboratories, Center for Devices and Radiological Health, U.S. Food and Drug Administration, Silver Spring, MD 20993, USA; rucha.natu@fda.hhs.gov (R.N.); suvajyoti.guha@fda.hhs.gov (S.G.); ahmad.dibaji@fda.hhs.gov (S.A.R.D.)

**Keywords:** microfluidics, spiral channel, inertial separation, particle focusing, sorting, flow performance testing

## Abstract

The development of new standardized test methods would allow for the consistent evaluation of microfluidic medical devices and enable high-quality products to reach the market faster. A comprehensive flow characterization study was conducted to identify regulatory knowledge gaps using a generic inertia-based spiral channel model for particle sorting and facilitate standards development in the microfluidics community. Testing was performed using 2–20 µm rigid particles to represent blood elements and flow rates of 200–5000 µL/min to assess the effects of flow-related factors on overall system performance. Two channel designs were studied to determine the variability associated with using the same microchannel multiple times (coefficient of variation (CV) of 27% for Design 1 and 18% for Design 2, respectively). The impact of commonly occurring failure modes on device performance was also investigated by simulating progressive and complete channel outlet blockages. The pressure increased by 10–250% of the normal channel pressure depending on the extent of the blockage. Lastly, two common data analysis approaches were compared—imaging and particle counting. Both approaches were similar in terms of their sensitivity and consistency. Continued research is needed to develop standardized test methods for microfluidic systems, which will improve medical device performance testing and drive innovation in the biomedical field.

## 1. Introduction

Microfluidic systems are becoming more common in biomedical applications [1,2], as evidenced by an increase in submissions of medical devices with microfluidics-based components to the U.S. Food and Drug Administration (FDA) [3]. The regulatory science of assessing microfluidic devices is just beginning to take shape. Tools such as standards and technical guidance will aid in product development, regulation, and process control. Standardized test methods inform guidance documents and regulations, which benefit device manufacturers by helping to establish clear and transparent expectations about the types of testing necessary for getting microfluidics products to market efficiently [4]. They are also important for creating consistency across device types and manufacturers and for ensuring device safety and effectiveness.

Currently, there are no dedicated regulatory standards or guidance for evaluating microfluidics-based medical devices. Existing test methods in the field have narrow scopes and specific applications that inhibit widespread acceptance [5,6,7,8]. The microfluidics community has only recently recognized the need for standards in microfluidics to translate laboratory systems to clinical use [9,10,11], though efforts toward the development of standards are underway [12,13]. To lay the groundwork for developing assessment tests, we chose a representative inertia-based particle sorting model as a case study for conducting different flow performance tests. The results from this study are meant to characterize device performance and identify technical hurdles associated with flow-based microfluidics platforms. The findings from this study are not to be interpreted as current FDA testing requirements or policy for microfluidic devices. Rather, this work is intended to support and encourage the development of performance testing strategies within the microfluidics community.

**Figure 1:**
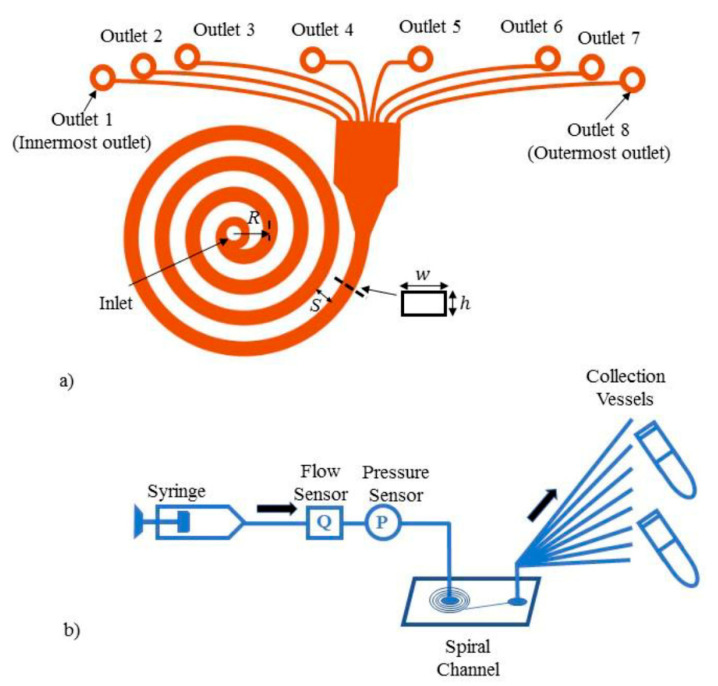
(**a**) Overhead view depicting the spiral microchannel flow model with 8 outlets, and (**b**) the easily reproducible test setup for the particle separation tests.

To choose a representative flow model for our case study, we considered several factors such as the design demand, the number of potential biomedical applications, the principle mode of cell or particle sorting, the potential impact of test parameters on overall performance, and the simplicity of the model. In part, due to the critical need for efficient cell sorting technologies in personalized medicine, a spiral inertia-based microchannel was chosen as the flow model for this study. A schematic of the spiral design is presented in Figure 1a. This design allows for membrane-free, label-free, and high-throughput biophysical cell separation, and the principle for size-based particle differentiation is well understood [14,15,16,17,18]. Archimedean spiral channels have been used to isolate different elements in blood such as circulating tumor cells (CTCs), HeLa cells, polymorphonuclear leukocytes (PMNs), mononuclear leukocytes (MNLs), and leukemia cells [15,19,20,21,22,23,24,25,26]. This study aims to provide a unique viewpoint to better predict microchannel performance, while considering issues that may arise when these types of devices are used in a clinical setting. There are several key factors such as microchannel fabrication, external device connections, device material, quality of the sample, the mechanism for pumping fluids, the fluid flow path, and human factors that may influence particle separation efficiency. To minimize the effect of the fabrication process, we have used a commercially available microchannel as our flow model. In addition, while many of these applications involve the single use of a disposable chip or cartridge, there may be some instances where multiple uses of the same microchannel are advantageous [27,28,29,30,31,32]. In some cases, only one component of the system is replaced after each use and the remaining components must demonstrate consistent performance over multiple uses [27,31]. When feasible, multiple uses of a shared flow pathway can make a system more economical, accessible, and sustainable. The methods used to analyze the measurands can also affect overall device performance. Image analysis [14,33,34,35] and flow cytometry [15,34,35] are commonly used analysis methods, but neither method has been standardized for microfluidic applications. Hurdles such as the ones described in this paper can hinder the translation of devices from concept to clinical use.

The objectives of this study were to: (1) understand the predictability of focusing behavior, also referred to as separation efficiency, for the spiral microchannel model; (2) study the particle separation technique when reusing a chip compared to single use of microchips; (3) predict and detect flow-related failure modes in spiral microchannel; and (4) explore different analysis methods for assessing microchannel performance.

## 2. Materials and Methods

Key parameters that affect the particle separation efficiency in an Archimedean spiral device include: channel geometry (e.g., shape, cross-sectional dimensions, and channel aspect ratio) [35,36], particle confinement ratio [7], Reynold’s number (Re), and the Dean number to quantify the cross-sectional vortices in curved channels [14,37]. In Table 1, we define key design attributes affecting the performance of the two spiral channel models used in this study (Design 1 and Design 2). The representative microfluidic channels used in this study are commercially available microchips for research purposes only (Fluidic 382, microfluidic ChipShop, Jena, Germany), made of Polymethyl methacrylate (PMMA) thermoplastic.

### 2.1. Test Fluid and Test Parameters

The test methods described in this study are meant to be simple and easy to reproduce. Thus, to improve test reproducibility, we used a glycerin-water surrogate fluid (18% glycerin solution in ultrapure deionized water) seeded with polystyrene beads as the test fluid instead of using a biological fluid that would vary from donor to donor. Details about the fluid properties, particle sizes, channel dimensions, and test conditions are provided in Table 2. The particle density intentionally matched the fluid density to minimize particle sedimentation during testing. A volume of 310 µL stock fluorescent particle solution (Spherotech, Lake Forest, IL, USA) was added per 10 mL of test fluid to achieve a particle concentration of at least 10^6^ particles/mL. Fifty microliters of Tween^TM^ 20 (Fisher Scientific, Portsmouth, NH, USA) surfactant were added to the mixture to minimize adhesion of particles to the channel walls. Two channel designs with different dimensions but similar aspect ratios (Design 1 and Design 2) were used to determine how the channel cross-sectional dimensions and confinement ratios impact particle separation efficiency. The test fluid was driven through the spiral microchannel at a pre-set, steady flow rate ranging from 200–5000 µL/min using a 10 mL syringe and a syringe pump (PHD ULTRA, Harvard Apparatus, Holliston, MA, USA), as illustrated in Figure 1b.

### 2.2. Test Setup

A schematic of the test setup used in all our experiments is provided in Figure 1b. Luer connectors were attached to the microchannel inlet and outlets. The interconnections were sealed using an adhesive and sealant (Loctite super glue and Dow Corning silicone 732, McMaster-Carr, Chicago, IL, USA) to prevent fluid leakage from pressures up to 300 kPa, a value which corresponds to the pressure in the main channel at the highest flow rate. Ideally, the pressure drop should be the same across each outlet, making the flow rate in each of the 8 outlet channels the same to prevent biases in particle focusing. The small diameter outlet extension tubes (0.007” ID PEEK plastic tubing, McMaster-Carr, Atlanta, GA, USA), with pre-calculated lengths, were connected to the outlet connectors to feed into the 8 collection vials for further particle concentration analysis. These extension tubes were connected at each outlet to compensate for any potential differences in lengths among the outlet channels of Designs 1 and 2 and to ensure the same pressure drop across each outlet.

The pressure drop, ∆P, in each channel outlet was calculated using the Hagen–Poiseuille’s equation [38],

(1) $$∆P=\frac{8\mu LQ}{\pi D_{h}{}^{4}}$$

where μ is the dynamic viscosity of the fluid, *L* is the length of the outlet channel, *Q* is the flow rate, and the flow is laminar. The pressure drop across the Design 1 chip ranges from 6 to 300 kPa for flow rates ranging from 200 to 5000 µL/min, respectively.

A total of 1.5 mL of test fluid seeded with particles was pumped through the microchannel at each flow rate (Table 2). The flow rate in the channel was measured using a flow-through sensor (MFCS-EZ, Lowell, Fluigent, MA, USA). A pressure sensor (Elvesys, Elveflow, Paris, France) was integrated into the flow system before the chip inlet to measure the pressure in the circuit over time. The flow rate sensor has an instrument error of <10%, and the pressure sensor has a reported accuracy of 0.2% of the maximum range. The limiting range of the pressure sensor is 103 kPa, and thus it was only used for tests up to 750 µL/min. Polytetrafluoroethylene (PTFE) tubing was used for the flow connections (ID: 0.5 mm, OD: 1.0 mm). At least 50 µL of sample were collected from each outlet and analyzed to determine the distribution of different size particles across the width of the channel.

To assess the effects of multiple uses of the same microchannel as well as chip-to-chip variability, intra- and inter-chip testing were conducted in triplicate (n = 3). For these tests, the same testing protocol was used for each replicate. To simulate a multiple-use scenario, the fluid-contacting surfaces of the channel were wetted and cleaned by flushing with 1.5 mL of distilled water and surfactant at 1500 µL/min between each test run.

The highest flow rate of 5000 µL/min could not be tested in Design 2 due to its smaller dimensions and higher corresponding backpressure. Leaking was observed at the inlet connection when the channel pressure exceeded 350 kPa. Additionally, the ability to focus different size particles simultaneously was only studied in Design 2 by mixing different particle sizes in a single solution. Three hundred ten microliters each of 6- and 16-µm stock particle solutions were added to 10-mL test fluid to assess the impact of particle-to-particle interactions at 750 and 1500 µL/min. Multiple particle sizes were not studied at 200 µL/min, because the 6- and 16-µm particle streams do not focus at this low flow rate.

### 2.3. Failure Modes

To understand the effect of contamination, different types of blockages—progressive and complete outlet—were introduced in the channel. Blockages of varying degrees can occur due to the unwanted presence of fibers, dust particles, particle aggregates, and bubbles in the system. To simulate such events, we created blockages by systematically introducing heterogenous fibers scraped from a representative source (i.e., delicate task wipes) into the channel inlet along with the test fluid via syringe. While the contaminants and excess particles were introduced into the flow in a consistent manner, the precise location and extent of the resulting progressive blockage was not controlled in this study. During fluid flow, the fibers settled at the entrance of some outlets over time to form what is referred to here as progressive blockages. Complete outlet blockages, in contrast, were created in a more consistent way by completely sealing only Outlet 3 with an adhesive. By acutely blocking all fluid flow through a single outlet, the channel only had 7 functioning outlets among which the particles could distribute. The effects of channel clogging were studied using only Design 1.

### 2.4. Particle Analysis Methods

A particle counter (Z2 Coulter Counter, Beckman Coulter, Indianapolis, IN, USA) was used to quantify the initial and exiting concentrations of particles. For particle counting, we set the acceptance thresholds to be the defined particle size ±2 µm, ensuring that the presence of large particle aggregates or contaminants would not lead to miscounts. Due to the lower measurement limits of the particle counter, a slightly narrower range of 1.5 to 4.0 µm was used when counting the 2 µm particles. Fifty microliters of the collected sample were mixed with 10 mL ISOTON II Electrolyte solution, which was used as a diluent in the particle counter. Particle counts were made on two samples from the same aliquot, and the average value was reported. Unless otherwise specified, the test results reported in this paper employed the particle counting method of analysis.

Image analysis is another common method for analyzing test results. We used image analysis to only assess focusing of single size particles, as opposed to mixtures of different size particles, to avoid potential limitations with particle size recognition. For image analysis, the microfluidic system was mounted on a manually adjustable microscope platform. Videos were recorded at 15 frames per second (fps) for 100 s using a microscope (Nikon T2 Eclipse microscope, Nikon Corporation, Tokyo, Japan) in conjunction with a compatible camera (Imager Pro X, LaVision, Ypsilanti, MI, USA). Up to 100 images were taken for every test condition to acquire 150 particles for each analysis. The image data were analyzed using ImageJ (National Institutes of Health and LOCI, Madison, WI, USA) to determine the position with respect to the channel walls of at least 150 particles in total and the separation efficiency across different outlets. Each image was post-processed to enhance contrast such that the particles were clearly visible as distinct entities against the background. Only the pixelated structures with light intensities 80% higher than the background intensity were defined as particles. The size range for the particles was specified so that entities falling outside the anticipated size range of ± 2 µm were not counted. The particle circularity acceptance criterion was specified to an allowable range of 0.9 to 1.0, ensuring that only in-focus round objects were counted as valid particles. Particle circularity is measured by a plugin in ImageJ using the equation circularity = *4π × [Area][Perimeter]*^2^ with a value of 1.0 indicating a perfect circle [39]. Based on the difference in intensities of the particles and background, the ‘analyze particles’ function in ImageJ was used to identify and screen for particles meeting these acceptance criteria. The particle counts, along with x- and y-coordinates of each individual particle, were recorded for each image. The width of the particle stream was determined to the nearest 15 µm based on particle positions. Note that image analysis was performed only for Design 1. In the end, the two methods of analysis—image analysis and particle counting—were compared to better understand their respective sensitivities and limitations.

### 2.5. Data Analysis

Error bars indicate the standard deviation (SD) from the mean, albeit only for three replicate test runs (n = 3). It should be noted that the average of two particle counts was determined for each test condition. For interpreting the consistency of the tests, we calculated the coefficient of variation (%CV = SD/mean × 100%) at each outlet for a specific particle size and flow rate. A higher coefficient of variation indicates greater variability in test results. One-way ANOVA tests allowed us to compare the empirical predictions and particle separation data for statistical significance (*p* < 0.05).

Skewness, which refers to the amount of shift in the data relative to a normal distribution, was calculated to quantify the distribution of particles across the width of the microchannel. A positive skewness value indicates particle focusing to outlets closer to the inner wall (Outlets 1–4), whereas negative skewness points to particle focusing to outlets closer to the outer wall of the channel (Outlets 5–8). Kurtosis, which indicates the quality of distribution or the sharpness of the distribution peak, was also calculated to quantify the overall spread in particle focusing. A higher kurtosis value corresponds to a more narrowly focused particle stream, or better focusing behavior of the microchannel.

## 3. Results

### 3.1. Particle Size and Flow Rate

**Figure 2:**
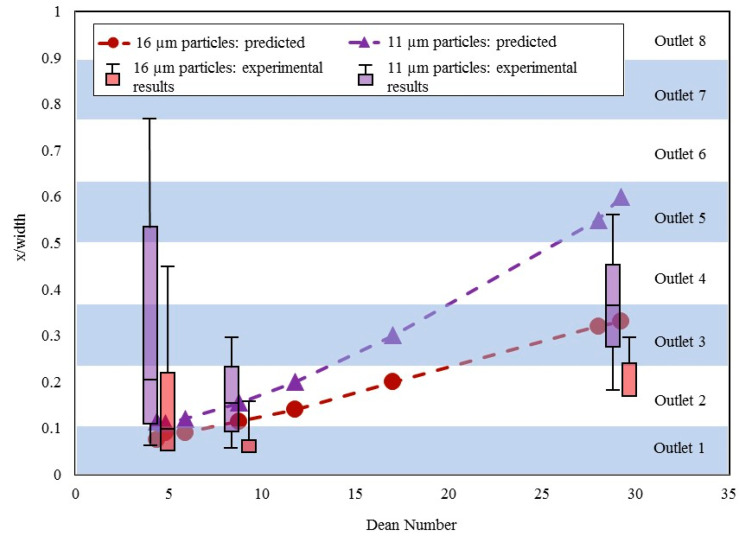
The predicted particle focusing behavior compared to experimental results of Design 1 for 11- and 16-µm particles [15,21,41]. The box-and-whisker results in this figure represent the median, 1st quartile, and 3rd quartile of the particle stream. The error bars include the 5th- and 95th-percentile of the particles with respect to the discrete outlet in which they were recovered.

The ability of a spiral channel to separate particles or focus them along a streamline depends on factors such as particle size, flow parameters, and channel geometry. Although there is no consensus flow model used by the microfluidics community to study particle focusing, several research groups have investigated the physics behind particle focusing for different flow rates and types of particles [14,15,21,23,24,33,40,41,42]. To validate our test results, we reviewed published studies involving spiral microchannels with similar geometries to Design 1. The predicted particle focusing behavior, which was interpolated from empirical data, is plotted as a function of *De* and particle size in Figure 2. The distance from the wall to where the particles focus is determined by normalizing the focusing distance (x/width) with respect to the Dean number and is shown by the primary *y*-axis. Here, Outlet 1 is the outlet closest to the inner wall of the spiral. Although we studied the focusing behavior of Design 1 using particles with diameters ranging from 2 to 16 µm to represent blood elements, only the 11- and 16-µm particles met the criterion of having a confinement ratio greater than 0.07, which is a reported threshold for inertial focusing to occur [43]. Therefore, plots for only those two size particles are shown in Figure 2. For a Dean number of 8.8, the 11- and 16-µm particles should collect primarily in Outlets 1 and 2. This can be determined by drawing a horizontal line from the corresponding data point on the plot to the corresponding outlet on the right.

While researchers have used similar flow models to Design 1 in previous studies [15,21,41], particle separation results have not been published using microchannel geometries comparable to Design 2. We used an unpaired two-tailed Student’s t-test to test the null hypothesis that the results from our experiments would be similar to the results predicted based on the literature [15,21,41]. The test confirmed that there was no statistical difference between the predicted and experimental results (*p* > 0.05), though the test results deviate from the predictions at the lowest and highest Dean numbers, as seen in Figure 2. For example, the 11-µm particles are predicted to collect primarily in Outlet 5 as the *De* number approaches 30, but the experimental results show that the particles focus in Outlets 3 and 4. Similarly, while we predicted that the 11-µm particles would focus in Outlets 1 and 2 at the low Dean number of 4.4, the experiments showed them more widely distributed across Outlets 1–6.

**Figure 3:**
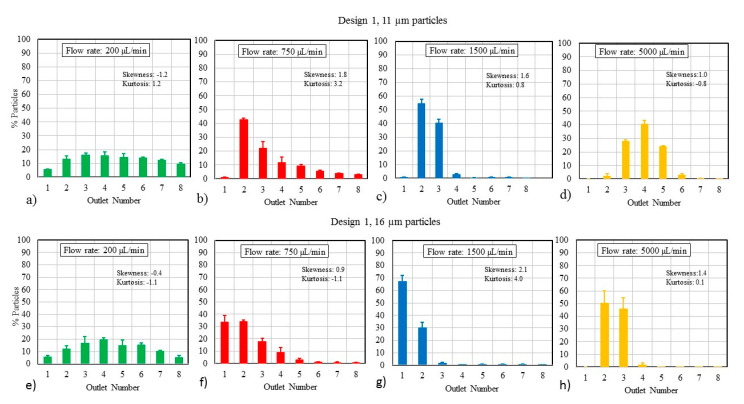

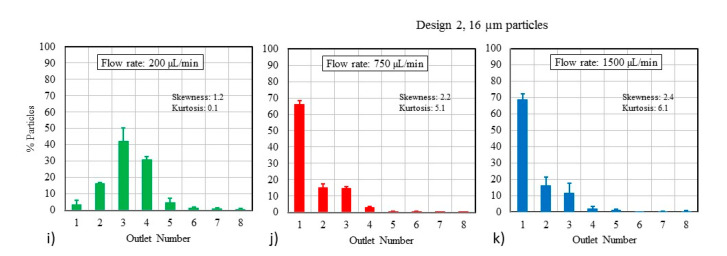
(**a**–**d**) Particle distributions for Design 1 using 11-µm particles at different flow rates, showing focused particle streams shifted away from the inner wall with an increase in flow rate. (**e–h**) Particle distributions using Design 1 for 16-µm particles at different flow rates. The 16-µm particles focus closer to the inner wall compared to the 11-µm particles. (**i–k**) Particle distributions for 16-µm particles at different flow rates using Design 2.

Design 1 was assessed over its full operating range at flow rates of 200, 750, 1500, and 5000 µL/min, which correspond to *De* values of 1.2, 4.4, 8.8, and 29.2, respectively. The focusing ability of the channel, which should directly correlate to separation efficiency, is demonstrated in Figure 3a–d for 11-µm particles and in Figure 3e–h for the 16-µm particles. The sum of all particles accounted for in the outlets adds up to 100% in each case.

The effect of channel dimensions on the performance was also studied. Figure 3i–k displays the impact of channel size on particle focusing (n = 3) by using Design 2. At 1500 µL/min, both Designs 1 and 2 are effective at focusing 16-µm particles, as seen in Figure 3g,k. However, for lower flow rates, Design 2 exhibits better focusing behavior than Design 1, due to a higher confinement ratio (Table 1). The higher confinement ratio caused an increase in lift force on particles and concomitant focusing towards the inner wall. This is suggested by the higher kurtosis value for Design 2 of 6.4 at 1500 µL/min, compared to a kurtosis of 4.0 for Design 1. Therefore, a microchannel with a higher confinement ratio design is likely to be more effective at separating large particles such as CTCs at relatively low flow rates.

**Figure 4:**
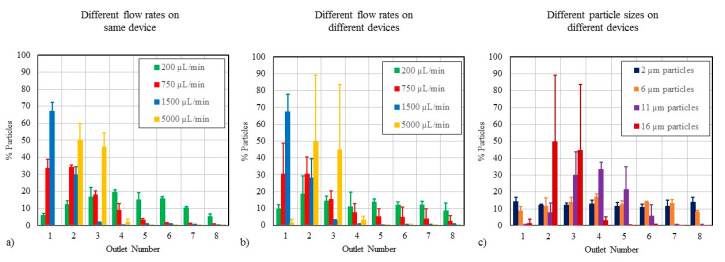
(**a**) Reusability associated with using the same microchannel multiple times (n = 3 repeats) for Design 1 with 16-µm particles. (**b**) Single-use testing of three replicate microchannels (Design 1) with 16-µm particles. (**c**) Single-use testing at 5000 µL/min for different particle sizes using Design 1.

While most microfluidic chips used for point-of-care diagnostics or therapeutic applications are likely to be single-use disposables, some complex devices contain multiple-use microfluidic components. Thus, we investigated how single-use versus multiple-use scenarios affect particle focusing efficiency in triplicate (n = 3). First, we examined the agreement in test results obtained using the same test method on a single microfluidic chip (i.e., multiple uses of the same chip), and then, we assessed the overall variability when multiple chips are used for the same application (i.e., single uses of replicate chips). The coefficient of variation (% CV) was calculated for each test condition and used to assess the variability in the results. Figure 4a shows how reusability affects the focusing of 16-µm particles at different flow rates, and Figure 4b shows single-use chips tested at different flow rates for the same size particle. Reusing a single chip multiple times produced more consistent results (27% CV) than the chip-to-chip variability of 80% CV at the highest flow rate, but the consistency in reusing the same chip was worse than the 4–10% CV reported for other microfluidic applications [44]. For the 5000 µL/min condition in Figure 4b, the particles appear to focus somewhere between Outlets 2 and 3, creating a situation where subtle changes to the test system may direct most particles toward one outlet or the other, which explains the large error bars.

Figure 4c shows the chip-to-chip variability for different particle sizes at 5000 µL/min. The 6-µm particles generated more consistent particle distributions (16% CV) than the larger 11-µm particles (60% CV). Further, the variability in chip-to-chip tests decreases as flow rate is reduced. At 1500 µL/min, the particle focusing tests with 16-µm particles had a 27% CV.

The results when using Design 2 at 1500 µL/min were consistent (18% CV) for the 16-µm particles. Design 2 was comparable to Design 1 in terms of reusability and single-use applications at all flow rates (data not shown); thus, changes to the microchannel cross-sectional dimensions and confinement ratio did not have much impact on the variability in particle distribution.

### 3.2. Particle Mixtures

For simplicity, most tests presented in this paper were performed using only one particle size at a time. However, for point-of-care applications, blood is likely to be the most common fluid passing through the microfluidic channel. Since blood contains elements of different sizes, we also studied the ability of a microchannel to separate and focus particles of different sizes simultaneously. Two different size particles (6 and 16 µm) were introduced in the channel at the same time to assess the focusing behavior of the larger 16-µm particles in the presence of smaller particles. The skewness and kurtosis values for the 16-µm particles when run with no other particles were 2.4 and 6.1, respectively, at 1500 µL/min. When placed in a heterogenous mixture of particles, these statistical values for 16-µm particles were similar, at 2.6 and 7.2, respectively. This indicates that the presence of other size particles does not significantly affect the particle focusing behavior, though the overall particle concentrations regardless of particle size are likely to impact particle focusing efficiency.

### 3.3. Flow-Related Failure Modes

According to a recent survey conducted in the microfluidics community, the unwanted presence of bubbles and clogging have been identified as two of the most common failure modes in microfluidic devices [45]. While the physics of such contaminants have been well-studied [46,47,48], limited work has been done to explore how bubbles and clogging affect microfluidic device performance [49,50]. Thus, we created a worst-case test condition to simulate these failure modes and evaluate how they affect particle separation. We artificially induced the following failure modes: (a) complete outlet blockage, created by fully blocking one outlet, and (b) progressive blockage, referring to a gradual blockage of one or more outlets due to the collection and accumulation of debris or particles over time.

**Figure 5:**
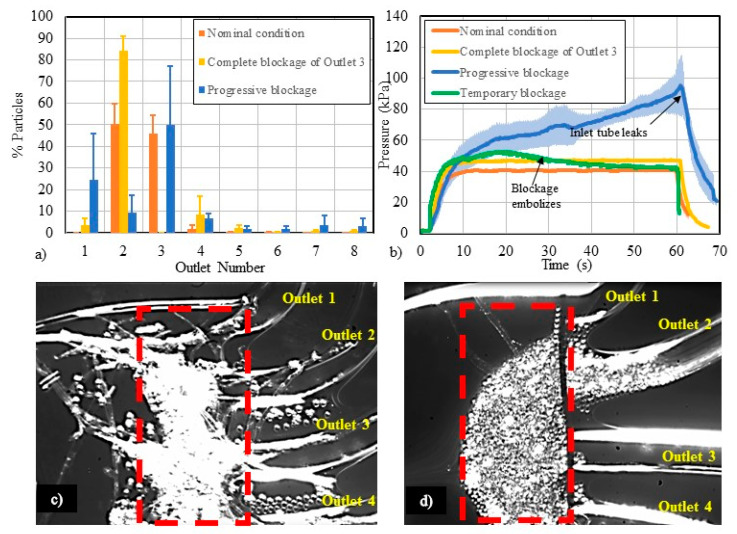
(**a**) The particle distribution profiles of 16-µm particles collected at 5000 µL/min due to different types of channel blockages. Error bars represent standard deviations from triplicate tests. (**b**) A pressure study for different cases of channel contamination/blockages was conducted at 500 µL/min. (**c**) Contaminants in a microchannel (fibers) can impact device performance and predictability. (**d**) Particle blockages can span multiple outlets, affect device performance, and possibly embolize.

Figure 5a displays particle focusing using 16-µm particles at 5000 µL/min under nominal and blocked-channel conditions. Under the nominal, unobstructed condition, most particles collected in Outlets 2 and 3. When a complete outlet blockage was created by blocking all flow to Outlet 3, most of the particles were redirected and recovered from Outlet 2, demonstrating a substantial shift in the separation efficiency. A progressive blockage was created in the channel as fibers accumulated at the proximal end of the outlets. These fibers trap particles to create an expanding blockage in the channel. It became more difficult to predict the particle focusing behavior when a progressive blockage was introduced into the system (Figure 5a).

Figure 5b shows the pressure measurements made in the main channel of Design 1 under nominal conditions, as well as with different types of blockages. When Outlet 3 was completely blocked, the pressure in the circuit increased by about 10%. For a progressive blockage of an outlet, the pressure in the main channel increased above the nominal flow condition. As particles begin to accumulate, backpressure builds within the channel. The pressure in the main channel elevated up to 250% of the nominal pressure, which increased the likelihood of leakage at the inlet chip-to-tubing interconnection. An image of this type of blockage is shown in Figure 5c. Another interesting phenomenon observed in this study was the temporary blockage caused by particle accumulation and subsequent embolization. Particles can accumulate at the entrance to the outlet and create backpressure in the channel. An example of particle accumulation can be seen in Figure 5d, where particles accumulated at the entrance to the outlets, creating a partial blockage of flow spanning Outlets 2–4. Here, the pressure in the main channel built up within 10–20 s, as particles accumulated near the outlets (green line in Figure 5b). After the pressure increased to about 33% above the nominal condition, the particles embolized and were flushed through the outlets. After the occlusion embolized, the pressure gradually decreased until the nominal steady flow condition was reestablished.

### 3.4. Study of Analysis Methods

**Figure 6:**
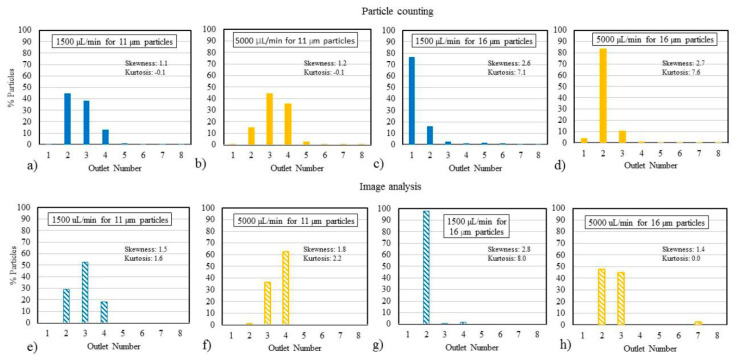
(**a**,**b**) Particle distributions using the particle counting method for 11-µm particles at flow rates of 1500 and 5000 µL/min, respectively. (**c**,**d**) Particle distribution profiles using the particle counting method for 16-µm particles at 1500 and 5000 µL/min, respectively. The high kurtosis value indicates focusing of particles across a narrow range. (**e**,**f**) Particle distributions using image analysis for 11-µm particles at flow rates of 1500 and 5000 µL/min, respectively. (**g**,**h**) Particle distribution profiles using image analysis for 16-µm particles at 1500 and 5000 µL/min, respectively.

Figure 6 shows particle focusing plots for 11- and 16-µm particles at 1500 and 5000 µL/min using particle counting (Figure 6a–d) and image analysis (Figure 6e–h). The particle distributions generated through image analysis show similar focusing behavior to the particle counting analysis at the same test conditions. Based on a one-way ANOVA, the two analysis methods are not statistically different.

## 4. Discussion

### 4.1. Particle Focusing Behavior

The experimental results were not statistically different from the predictions. However, certain discrepancies were observed between the two datasets, particularly at higher *De* numbers. Subtle differences in channel geometry could lead to such discrepancies. For instance, the microfluidic channels used to make the predictions had slightly different channel heights of 110, 130, and 140 µm, though the channel width was consistent in all three cases at 500 µm [15,21,41]. In comparison, Design 1 in this study has a channel height of 120 µm and a width of 500 µm. The differences in channel geometries directly affect the confinement ratio and accuracy in predicting particle focusing behavior. The locations of the particle streams relative to the channel walls was required to interpolate the predicted particle sorting behavior of similar microchannels for comparison [15,21,41]. This comparison was made because the particle sizes and channel dimensions were similar to the conditions used in our study. The positional data were linearly interpolated for the channel dimensions of Design 1 to predict sorting of 11- and 16-µm particles at specific *De* numbers. The particle distributions measured directly via particle counting were compared to the interpolated predictions in Figure 2 to gain a better understanding of the predictability of inertial-based particle separation testing. The discrepancies between the experimental results and predictions show the importance of validating particle separation data in microchannels via bench testing. While predictions based on empirical data can provide insight into device performance, subtle changes to a simple microchannel geometry were shown to impact particle sorting in this case.

We observed several trends based on our inertia-based particle separation testing: (a) focusing behavior strongly depends on flow rate, (b) superior focusing behavior was achieved at 1500 µL/min for both particle sizes, (c) at larger flow rates, most of the particles were collected primarily through two adjacent outlets, though the location of those outlets varied, (d) larger particles were less susceptible to changes in flow rate compared to the smaller particles, and (e) as flow rate increases, the particles focus farther away from the inner wall. These trends agree with the trends observed in the literature [15,21,41]. We hypothesize that differences in material properties (i.e., PMMA was used here, while elastic PDMS chips are commonly used in research labs), disparities in test conditions (e.g., actual flow rate versus reported flow rate, actual particle size versus reported size, and tolerance of channel dimensions) and a linear interpolation of *De* when the physics are non-linear have contributed to the differences between the predictions and experimental results [51].

It is important for medical devices to perform consistently and reliably. The consistency when using a single chip multiple times was better than the chip-to-chip variability in terms of the coefficient of variation. Larger variability in test results may be indicative of a greater likelihood of false positive and false negative readings. The particle separation tests became less consistent as the particle size increased. As expected, the variability in test results increased with increasing flow rate, such that there was a tradeoff in terms of throughput and accuracy of the test results in spiral separation channels. A visual analysis of the particle streams confirmed large oscillations in particle focusing at the highest flow rate of 5000 µL/min compared to 1500 µL/min. Therefore, a positive test result with a high-throughput microfluidic device may necessitate additional tests for confirmation. It should be emphasized that the coefficient of variation values presented here are based on a small sample size of three, whereas the number of samples to be used for determining variability during scale-up would be significantly higher to achieve meaningful statistical power. Therefore, the % CV in finalized spiral device designs may turn out to be different from these reported values.

Some potential sources of error that can greatly impact device effectiveness include the quality of microfluidic interconnections, the pressure fluctuations and pulsatility in the pumping system [52,53], particle buoyancy, the fluid-structure interface, and other fluid-contacting components such as the inlet tubing and connectors. For multiple-use or long-term use devices, the effect of residue buildup in the channel, material degradation, or particle accumulation can cause device performance to deteriorate over time. As previously mentioned, the connections for the inlet and outlet tubes were secured using an adhesive to prevent leaks. The quality of these interconnections can be determined by user skills or manufacturing processes, all of which can contribute to the chip-to-chip variability. These types of fabrication issues need to be addressed when upscaling device production for commercial use.

### 4.2. Effect of Using Particle Mixtures

Use of particle mixtures in spiral channels represents a clinical scenario in which a heterogenous cell sample passes through a device for separating cells based on size. In this study, we did not find any significant difference in the focusing behavior of the particles in the presence of other particle sizes. However, studies performed with clinical samples using other types of microfluidic particle separation devices found particle trajectories to be impacted by concentration [34]. Higher particle concentrations can result in a crowding effect that prevents particles from focusing in a small region due to limited available space. At a concentration threshold, particle-to-particle interactions caused by an overall higher concentration of particles in solution inhibit the ability and effectiveness of the device to focus particles based on size. Further tests need to be conducted to establish the threshold at which particle-to-particle interactions within particle mixtures begin to impact the focusing behavior in a microfluidic device. To compensate for overly concentrated particle solutions, samples may need to be diluted accordingly to achieve optimal test results. The need for sample dilution is a limitation of many microfluidic systems. However, it appears that the particle concentrations used in this study fall below that critical threshold for impacting particle focusing behavior.

### 4.3. Study of Flow-Related Failure Modes

This study shows that channel blockage can cause microfluidic system failure and inaccuracy. Medical device failures are largely considered to be underreported, perhaps more so for diagnostic devices that may not necessarily cause direct harm to a patient. In point-of-care diagnostic applications, microfluidic devices can be subjected to many types of known and unknown contaminants. The presence of dust particles or fibers which could be introduced during handling are some such examples. For plug-and-play types of devices, the human factors associated with making leak-free and bubble-free connections can affect flow performance. Blockages or the introduction of contaminants in a micro-channel can affect the derived output and have serious clinical implications. For example, if a similar channel design to the one used here is indicated for detecting CTCs in blood samples and a partial blockage forms across Outlets 1–3 causing the CTCs to be unexpectedly diverted to Outlets 4 and 5, the microchannel may inaccurately determine that the patient is cancer free. Such a false negative result may delay time-sensitive treatment for a patient. Precautions should be taken by the community to minimize contamination and user error so as not to affect the test results. To reduce the risk of device failures, preventative measures can be taken, such as adding filters or bubble traps to the system, using anticoagulants, and diluting the test samples. In addition to these mitigation strategies, making multiple replicate measurements helps to reduce the likelihood of false positive and false negative results.

Along with visual detection, we found pressure measurements to be a sensitive detection method for monitoring the operating status of a microfluidic system. The pressure in the fluid circuit rises depending on the extent of blockage in the channel. For example, for a temporary blockage the pressure may increase by about 33%, but the pressure could return to the baseline level when the blockage embolizes and the particles are flushed through the channel. In the case of a progressive blockage, the pressure increased up to 250% of the nominal pressure in this study. All types of blockages can affect the fluid and particle flow paths in the channel, and thus the overall device performance is compromised. Given the sensitivity of local pressure measurements to different types of blockages, pressure measurements may offer an alternative real-time approach for routine monitoring of the channel status within a diagnostic or therapeutic device.

### 4.4. Differences in Analysis Methods

Particle counting, which relies on the Coulter principle [54], has been a gold standard for quantifying clinical labs for decades. However, with a growing need for point-of-care diagnostics and with patients and physicians depending on immediate, accurate test results, we have explored the feasibility of particle imaging as an alternative means to particle counting. Imaging is quicker and potentially more informative as it provides temporal-, position-, shape-, and sized-based information of particles. Imaging techniques have been widely used for analyzing the performance of spiral channels [15,36]. While the particle counting and image analysis methods generate similar test results, the reported focusing behaviors were slightly different depending on the analysis method used. One difference was the number of particles needed for analysis—the number of particles sampled in particle counting exceeded 1000 particles compared to only 150 particles captured for accurate image analysis. The detectability of particles in image analysis is dependent on the ability to distinguish light intensity of particles from the background, whereas the Coulter counter measures particles in terms of a change in electrical impedance as the particles pass through a narrow aperture. Additionally, the images were acquired earlier in the channel, before the flow enters the expansion region leading to the proximal end of the outlets. Particle counting was based on samples collected in vials that were attached to the distal end of each outlet tubing.

A more comprehensive study is necessary to determine which analysis method is superior for determining the effectiveness of particle focusing and separation devices. An image analysis may be problematic for a clinical test involving opaque fluids like blood. In such cases, cells may require an additional labeling step to include fluorescent tags for optimal detection of target elements.

### 4.5. Limitations

While we believe that this study of an inertia-based sorting microchannel is useful for stimulating regulatory science in the emerging field of microfluidics, the study does have several limitations. First, only two microchannel designs were studied here and therefore extrapolation of implications to other channel dimensions, microchannel designs, materials, medical devices, and flow-related applications should be done with caution. Second, the fluid used for these experiments is a blood mimicking fluid which is a particulate mixture of glycerin and water. Thus, while the test fluid can be easily and reproducibly formulated, this study does not necessarily replicate failure modes that would be expected when using diluted or whole blood. In addition, the rigid polystyrene beads we used in this study were not deformable cells (e.g., red blood cells), and thus the resulting particle focusing behavior may be impacted by the particle properties being sorted. Further testing is needed to determine how the test fluid impacts particle or cell separating efficiency in microchannels. Moreover, the microchannel used in this study is simplified, meaning that additional system components such as interconnections will need to be modified to better represent a medical device. For example, in the case of a medical device used to separate cells based on size, higher specificity, a continuous elution mechanism and a higher number of replicate trials would be expected to establish the repeatability and reproducibility of device outputs. The manually operated batch systems and manual connections with adhesive that were used in this study are unlikely to represent a commercial medical device. Despite these limitations, we anticipate that the issues discussed in this study provide insight into different technical issues to consider when transforming a lab-based system into a commercial medical device.

## 5. Conclusions

Much of the current research on microfluidic-based medical devices focuses on demonstrating proof-of-principle for specific assays, while the reliability of end-devices operating under clinically relevant conditions is not always considered in the early stages of product development [55]. With more microfluidic devices entering the market, it is imperative to develop new assessment tools, standards, and streamlined approaches for evaluating safety and effectiveness. Standardized models can help to optimize microchannel design, operating ranges, sample collection, interconnections between the microfluidic channel and other system components, the analysis method, and test consistency.

To support this effort in developing standard test methods, we performed simple bench tests to investigate the impact of channel dimensions, flows, and reliability parameters on a generic spiral microchannel model. A summary of the major findings, along with the potential clinical significance, is reported in Table 3. Several design variables and failure modes commonly seen in microfluidic systems were found to substantially impact the particle separation/focusing behavior of the microchannel. The two analysis methods used in this study, image analysis and particle counting, produced comparable results. Reusing a single microchannel several times produced more consistent results than using a new microchannel for each test. Given the diversity in commercialized microfluidic applications, the importance of these factors will vary on a case-to-case basis. Although a spiral microchannel was used here as a case study of particle sorting, further testing with different channel designs needs to be performed to determine the extent to which aspects of this study (e.g., microchannel reusability, effect of channel dimensions on flow performance, and pressure monitoring) translate to other microfluidic systems and applications. Inertia-based cell sorting is a promising application for microfluidic medical devices, but we hope to eventually identify commonalities and trends among other microfluidic device types for which standard fluid flow and flow sensing protocols can be applied. While much remains to be done in this area, we hope that more benchtop studies such as this are conducted over the entire operating range, including nominal and extreme test conditions, to help generate meaningful and reliable results and support clinical study data. Furthermore, mitigation strategies designed to identify and simulate failure modes will reduce the prevalence of false positive and false negative results.

We are encouraged by the huge potential for microfluidic medical devices, but we also recognize the immense challenge of standardizing aspects of a cross-cutting field with many different device types, intended uses, and biomedical applications. There is a need for standardized flow-related test methods to facilitate the commercialization and widespread use of emerging microfluidic-based devices, and the research presented here is meant as a small step toward that goal. Similar design-specific studies may help better characterize device performance and support, or reduce the reliance on, costly clinical trials. For the most promising test methods developed by individual labs, round-robin testing across multiple laboratories can be a valuable next step for efficient development of consensus, standard test methods. Currently, there are no standards specific to microfluidic medical devices, but we hope that regulatory science such as that described here will help to identify the knowledge gaps in medical device evaluation. Standardization often emerges from the discovery of commonalities identified for specific device types.

## Figures and Tables

**Table 1:** Important parameters for determining the separation efficiency of spiral microchannels.

Parameter	Equation	Design 1	Design 2
Aspect ratio (*AR*)	h*/w*; where h is channel height and w is channel width	0.24	0.27
Hydraulic diameter ($D_{h}$)	2hw(h+w)	193 µm	126 µm
Confinement ratio (δ)	dh; where d is particle diameter	0.01 (for 2 µm particles) 0.13 (for 16 µm particles)	0.02 (for 2 µm particles) 0.20 (for 16 µm particles)
Reynold’s number (*Re*)	ρuDhμ; where ρ is fluid density, u is fluid velocity and µ is dynamic viscosity	7 (at 200 µL/min) 168 (at 5000 µL/min)	16 (at 200 µL/min) 123 (at 1500 µL/min)
Dean number (*De*)	ReDh2R; where R is radius of curvature	1.2 (at 200 µL/min) 29.2 (at 5000 µL/min)	2.8 (at 200 µL/min) 21.2 (at 1500 µL/min)

**Table 2:** Test parameters investigated in this study.

Test Parameter	Description
Test Fluid	Glycerin (18% by volume) in ultrapure deionized water, with Tween 20 and concentrated particle solution (fluid density: 1049 kg/m^3^, dynamic viscosity: 1.6 × 10^−3^ Pa·s)
Surrogate Particles	2 µm, 6 µm, 11 µm, and 16 µm
Particle Concentration	10^6^–3 × 10^8^ particles/mL (for 16 µm particles at a concentration of 1.6 × 10^6^ particles/mL; for 11 µm particles ~ 3.3 × 10^6^ particles/mL; for 6 µm particles ~ 9.2 × 10^7^ particles/mL; for 2 µm particles ~ 2.7 × 10^8^ particles/mL)
Flow Rates	200, 750, 1500, and 5000 µL/min
Microchannel Material	Polymethyl methacrylate (PMMA)
Device Design 1	8 outlets, 4 rotations, 120 µm height, 500 µm width, 2 mm radius of curvature, 0.3 mm spacing between channels, and 56 mm channel length
Device Design 2	8 outlets, 4 rotations, 80 µm height, 300 µm width, 2 mm radius of curvature along the horizontal for the first loop, 0.3 mm spacing between channels, and 38 mm channel length

**Table 3:** Summary of the trends observed in this study and the clinical significances.

Test Parameter	Effect	Clinical Significance
Particle size ↑	Lift force on particles ↑, particle focusing shift → inner wall	A wide range of cell sizes can reduce the focusing effect and spread the particle streams, thus decreasing test sensitivity. Larger particle sizes are more impacted by Dean vortices. Particle-to-particle interactions are more prevalent with larger particles.
Channel hydraulic diameter ↑	Throughput ↑, channel pressure ↓, confinement ratio ↓	While processing time can be expedited by increasing the cross-sectional area of the channel, the separation efficiency may suffer. Particle-wall interactions are reduced in larger channels.
Flow rate ↑	Reynold’s number ↑, Dean number ↑, particle focusing shift → outer wall, particle focusing ↑ (except at highest flow rates)	Throughput increases at higher flow rates, as does pressure. However, leaks and fluctuations in flow can cause system failures.
Confinement ratio ↑	Lift force ↑, particle focusing shift → inner wall	Device effectiveness is increased for higher confinement ratios, and the particle streamline becomes more impacted by microchannel geometry.
Number of repeat uses of the same microchannel ↑	Uncertainty ↓	Increased testing reduces the uncertainty (e.g., variability) and causes fewer false positives, thus improving the reliability of the clinical results.
Channel blockage ↑	Channel pressure ↑, predicted particle focusing ↓	Blockages can cause sample loss, impact test sensitivity, and result in false positive/negative readings.
Detection method sensitivity ↑	Particle focusing ↑, reliability (i.e., repeatability and reproducibility) ↑	Better particle separation and detection methods will improve reliability, test sensitivity, and device performance.
